# Protective effect of alpha-lipoic acid and omega-3 fatty acids against cyclophosphamide-induced ovarian toxicity in rats

**DOI:** 10.14202/vetworld.2020.188-196

**Published:** 2020-01-27

**Authors:** Dhanya Venugopalan Nair, M. Usha Rani, A. Gopala Reddy, B. Kala Kumar, M. Anudeep Reddy, M. Lakshman, U. Rajkumar

**Affiliations:** 1 Department of Veterinary Pharmacology and Toxicology, College of Veterinary Science, P. V. Narasimha Rao Telangana Veterinary University, Hyderabad, Telangana, India; 2 Department of Veterinary Pathology, College of Veterinary Science, P. V. Narasimha Rao Telangana Veterinary University, Hyderabad, Telangana, India; 3 Department of Genetics and Breeding, ICAR-Directorate of Poultry Research, Hyderabad, Telangana, India

**Keywords:** alpha-lipoic acid, cyclophosphamide, omega-3 fatty acid, ovarian toxicity, oxidative stress

## Abstract

**Background and Aim:**

Cyclophosphamide therapy is known to be associated with the risk of female infertility as a result of ovarian toxicity. Alpha-lipoic acid (LA) and omega-3 fatty acids are known for their antioxidant and anti-inflammatory activities. The present study investigated the potential protective effect of alpha-LA, omega-3 fatty acids, and its combination against cyclophosphamide-induced ovarian toxicity in rats.

**Materials and Methods:**

Thirty rats were equally divided into Groups I, II, III, IV, and V. Group I was normal control, wherein the rats were fed with normal feed and water *ad libitum*. Group II served as cyclophosphamide-induced group, wherein the rats were injected with cyclophosphamide at 75 mg/kg through intraperitoneal route once a week to induce ovarian toxicity. Groups III and IV were treated with alpha-LA at the rate of 25 mg/kg and omega-3 fatty acids at the rate of 400 mg/kg, respectively, in parallel to cyclophosphamide induction as in Group II. Group V animals were coadministered with alpha-LA (25 mg/kg) and omega-3 fatty acids (400 mg/kg) along with cyclophosphamide induction as in Group II. The respective treatments were administered daily through oral route for a period of 30 days. Regularity of estrous cycle was evaluated by vaginal cytology. Post-treatment period, the animals were humanely sacrificed, and the blood samples were subjected to the estimation of follicle-stimulating hormone (FSH) and estrogen. The ovarian tissue was weighed and subjected to histopathology, transmission electron microscopy, estimation of decreased glutathione (GSH), and tumor necrosis factor (TNF)-alpha.

**Results:**

Rats treated with cyclophosphamide alone manifested irregularity in estrous cycle, increased FSH, and reduced estrogen levels. The ovaries showed decreased GSH and increased TNF-alpha concentrations. Histopathological and transmission electron microscopic analysis of the ovarian follicles revealed degenerative changes. Administration of alpha-LA and omega-3 fatty acids as well as the combination of both the treatments demonstrated significant normalization of the estrous cycle and antioxidant defense mechanism as well as ameliorated the hormonal profile and histological architecture of the ovarian follicles. However, appreciable synergistic efficacy of the combination therapy (alpha-LA+omega-3 fatty acids) with respect to the monotherapies was not observed in the present study.

**Conclusion:**

The efficacy of alpha-LA and omega-3 fatty acids against cyclophosphamide-induced ovarian toxicity could be attributed to its antioxidant and anti-inflammatory activities that prevented the oxidative damage to the ovaries caused by cyclophosphamide. Hence, our findings suggest that dietary supplementation of alpha-LA and omega-3 fatty acids in women receiving cyclophosphamide therapy could carry potential benefits in preventing cyclophosphamide-induced infertility in childbearing women.

## Introduction

Cyclophosphamide is a widely prescribed antineoplastic drug for various forms of malignant disorders. Unfortunately, premenopausal women exposed to cyclophosphamide are associated with an increased risk of developing premature ovarian failure (POF) characterized by early menopause and infertility [1-3]. Such a type of reproductive toxicity significantly reduces the quality of life in patients receiving cyclophosphamide [4]. Cyclophosphamide causes POF through oxidative damage of ovarian follicles followed by inflammation and apoptosis with subsequent depletion of ovarian reserve [5]. POF is diagnosed by a persistent increase in follicle-stimulating hormone (FSH) levels and decreased estrogen levels [6]. The current alternatives available to prevent the ovarian toxicity associated with cyclophosphamide include cryopreservation of oocyte or embryo and use of gonadotropin-releasing hormone (GnRH) agonists [7]. However, cryopreservation alternatives are expensive GnRH agonists expose the individual to various lifelong risks such as hormonal imbalance and loss of bone mineral density [8,9]. These disadvantages limit their use in clinical settings. The severity of the situation has created an unmet need for safe and effective alternatives for combating the infertility associated with cyclophosphamide. Nutritional pharmacology has paved the way through the development of various natural and synthetic antioxidants and few of these compounds are proven to be a potent resource to prevent infertility associated with cyclophosphamide [10-15].

Alpha-lipoic acid (LA) is a universal antioxidant and has been reported to be effective against various types of cardiovascular and neurodegenerative disorders [16-19]. It is a disulfide compound naturally occurring in the mitochondria and is often referred to as an ideal or universal antioxidant that protects the mitochondrial membranes against oxidative injury [20]. It has proven antioxidant activity in various disorders such as atherosclerosis, diabetes mellitus, multiple sclerosis, and dementia, wherein oxidative stress plays a major role in pathogenesis [21]. Alpha-LA is produced in mitochondria from octanoic acid. It is a cofactor of mitochondrial enzymes such as α-ketoglutarate dehydrogenase and pyruvate dehydrogenase and is involved in the production of acetyl coenzyme A, through the oxidative decarboxylation of pyruvate. *In vivo*, alpha-LA is reduced to dihydro-LA (DHLA), which neutralizes reactive oxygen species, chelates metal ions Fe^2+^, Cu^2+^ and Cd^2+^, and promotes regeneration of endogenous antioxidants such as glutathione (GSH), Vitamin E, and Vitamin C [22] and is reported to inhibit the release of pro-inflammatory cytokines [23]. In non-clinical species, alpha-LA has been effective in alleviating ovarian ischemia-reperfusion injury mediated by reactive oxygen species [24], malathion-induced mammary toxicity [25], as well as cyclophosphamide-induced oxidative stress in testes [26] and liver [27]. Alpha-LA supplementation has delivered promising results in oocyte maturation rate and ovarian antioxidant status [28].

Fish-derived omega-3 fatty acids, commonly referred to as “Fish oil,” consist of eicosapentaenoic acid (EPA), and docosahexaenoic acid (DHA). These precursors of certain eicosanoids are vital for various physiological functions and are known to reduce inflammation [29]. DHA plays a significant role in brain and behavioral development [30], whereas EPA is reported to inhibit phospholipase A2 enzyme leading to stabilization of membrane structure and subsequently prevents inflammation [31]. As mammals cannot introduce a double bond beyond the D-9 position in the fatty acid chain, omega-3 fatty acids are required to be supplemented externally to gain their benefits. Several studies have reported the beneficial effects of omega-3 fatty acids on reproductive system including regulation of ovarian folliculogenesis and estrous cycle, ovulation, embryo survival, and parturition [32-34]. Supplementation of omega-3 fatty acids is reported to improve the quality and functioning of the granulosa cells, oocytes, and embryos [35]. Fish oil has demonstrated effectiveness in preventing oxidative stress, reducing inflammation, and enhancing fertility [36,37]. It is reported that omega-3 fatty acids are highly sensitive to oxidation and hence its combination with alpha-LA would reduce the oxidation of omega-3 fatty acids and maintain the therapeutic efficacy [38]. Hence, the combination of alpha-LA and omega-3 fatty acids was considered as a part of this investigation.

As cyclophosphamide exerts its toxicity through oxidative damage, the present study was conducted to investigate the protective effect of two antioxidant compounds, i.e., alpha-LA and omega-3 fatty acids in preventing the cyclophosphamide-induced ovarian damage. To explore the possibility of synergism as compared to the monotherapies, the efficacy of alpha-lipoid acid and omega-3 fatty acid in combination therapy was also studied as a part of this investigation.

## Materials and Methods

### Ethical approval

The protocols adopted in this experimental study were approved by the Institutional Animal Ethics Committee (IAEC), College of Veterinary Science, Hyderabad, India and are in accordance with the Guidelines for the Care and Use of Laboratory Animals published by the US National Institute of Health (IAEC Approval No. 2/2017-SA/16-5-2017).

### Chemicals and kits

All the chemicals (for the preparation of reagents and buffers) were procured from Sigma-Aldrich, USA, Qualigens Pvt. Ltd., Mumbai, India, and HiMedia Pvt. Ltd., Mumbai, India. Alpha-LA and omega-3 fatty acids were procured from SRL Pvt. Ltd., Mumbai, India. Enzyme-linked immunosorbent assay (ELISA) kit for tumor necrosis factor (TNF)-alpha was procured from Krishgen Biosystems, India. ELISA kits for estradiol and FSH were procured from Calbiotech Inc., CA, USA

### Animals

The experimental study was conducted in healthy adult female Sprague Dawley rats of 9-11 weeks age and weighing about 150-200 g on an average. The animals were reared under uniform environmental conditions with a temperature range of 22±2°C in polypropylene cages under 12 h dark/light cycle and provided with feed and water *ad libitum* throughout the study. Before the initiation of the experiment, the animals were acclimatized for a period of 10 days.

### Grouping and experimental induction/treatment procedures

The experimental rats were categorized randomly into five groups, withsix rats in each group. Group I was normal control, wherein the rats were fed with feed and water *ad libitum*. Group II served as cyclophosphamide-induced group, wherein ovarian toxicity was induced in rats with cyclophosphamide at the rate of 75 mg/kg through intraperitoneal route once a week. Group III served as alpha-LA-treated group, wherein the animals were administered with alpha-LA at the rate of 25 mg/kg through oral route along with cyclophosphamide induction as in Group II. Group IV served as omega-3 fatty acids treatment group, wherein rats were administered with omega-3 fatty acids at the rate of 400 mg/kg through oral route along with cyclophosphamide induction as in Group II. Group V was administered with a combination of alpha-LA (25 mg/kg) and omega-3 fatty acids (400 mg/kg) along with cyclophosphamide induction regime as in Group II. The selection of doses for alpha-LA and omega-3 fatty acids was based on previously published efficacy studies [39-42]. The analysis for the efficacy of the above compounds comprised vaginal cytology, serum analysis for FSH and estradiol, tissue analysis of the ovaries for GSH (reduced GSH), and TNF-alpha followed by histopathology and transmission electron microscopy of the ovaries.

### Evaluation of estrous cycle by vaginal cytology

In the present study, seven consecutive estrous cycles (each cycle comprising 4 days) were monitored for a period of 28 days by vaginal smear test [43,44] taken early in the morning on a regular basis by pipette smear technique. To enable the procedure, the rat was held around the thorax, ventral surface uppermost with one hand while the hand holding the pipette was used to restrain the tail, to provide additional support, and to help prevent the animal struggling. A small amount of approximately 0.2 ml of 0.9% normal saline was introduced into the vagina with a disposable soft plastic pipette (with an internal tip bore of 1.5 mm). The tip of the pipette was pushed gently into the entrance of the vagina to a depth of 2-5 mm and the fluid was flushed into the vagina and back up into the pipette 2 or 3 times by gently squeezing and releasing the bulb of the pipette. A small amount of the cell suspension was then expelled onto a labeled glass slide. Slides were labeled with the female identification numbers. The smears were further stained by Giemsa’s stain and observed under light microscope. The length of each estrous cycle [45,46] and relative lengths of each phase of estrous cycle, i.e., proestrous, estrous, metestrous, and diestrous was calculated as per previously published methods [47]. For each animal, the number of hours for which each of the four stages of the cycle was recorded was converted into the number of days (dividing the number of h with 24).

### Serum analysis

Blood collection was performed from day 30 as and when the animals came into proestrous phase as it was reported that the hormones are elevated during this phase [48,49]. Feed was withdrawn 12 h before the blood collection. Rats were anesthetized by ketamine (100 mg/kg) and xylazine (10 mg/kg) and blood was collected through retro-orbital plexus. Serum samples were separated from the blood by centrifugation and subjected to the estimation of FSH and estradiol. Measurement of FSH and estradiol levels was performed according to the standard protocols provided by Calbiotech Inc., CA.

### Ovarian tissue analysis

Post-blood collection, the rats were euthanized with carbon dioxide. The ovarian tissues were excised, trimmed from the surrounding adipose tissue, and washed gently in cold isotonic saline. A part of ovarian tissue was kept in neutral buffer formalin and glutaraldehyde and further processed for histopathology and electron microscopy, respectively. For histopathology, the tissues were processed and stained with hematoxylin and eosin (H and E) stain as per previously published methods [50]. The remaining tissues were homogenized (1:5, w/v) in chilled phosphate buffer using Polytron homogenizer and kept at −80°C until for the estimation of TNF-alpha and GSH. Estimation of GSH was performed as per previously described method [51]. The amount of TNF-α in the ovarian tissue was determined by ELISA kit obtained from Krishgen Biosciences Inc.

For transmission electron microscopic studies, small samples of ovaries were transferred to vials and fixed in 2.5% glutaraldehyde in 0.1 M phosphate buffer (pH 7.2) for 24 h at 40°C and washed with phosphate-buffered saline for 2 times each 45 min, then post-fixed in 1% aqueous osmium tetroxide for 2 h, later washed with deionized distilled water for 4 times each 45 min. After the post-fixation, samples were dehydrated in series of graded alcohols, infiltrated and embedded in Araldite 6005 resin or spur resin [52], and incubated at 800°C for 48 h for complete polymerization. Ultrathin (60 nm) sections were made with glass knife on ultramicrotome (Leica Ultracut UCT-GA-D/E-1/100), mounted on copper grids, and stained with saturated aqueous uranyl acetate and counterstained with Reynolds lead citrate and were observed at various magnifications [53] under a transmission electron microscope (Model: Hitachi, H-7500, Japan).

### Statistical analysis

The data were subjected to statistical analysis by applying ANOVA using the Statistical Package for the Social Sciences (SPSS) version 21 (IBM, USA). Differences between means were tested using Tukey’s *post hoc* test and the significance was set at p<0.05.

## Results

### Alpha-LA and omega-3 fatty acids regularized estrous cycle in cyclophosphamide-induced rats

**Figure-1:**
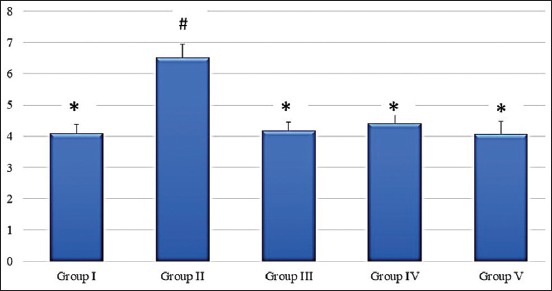
Graph showing average length of estrous cycle (days) in different groups. Values are mean±standard error (n=6). Means with similar symbols do not differ significantly. Group I: Normal control group; Group II: Cyclophosphamide-induced control group; Group III: Alpha-lipoic acid-treated group; Group IV: Omega-3 fatty acids treated group; Group V: Alpha-lipoic acid+omega-3 fatty acids treated group.

**Figure-2:**
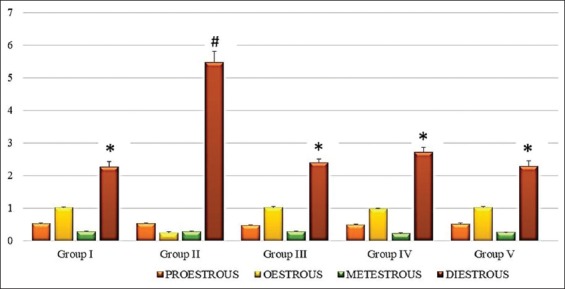
Graph showing average length of each phase estrous cycle (days) in different groups. Values are mean±standard error (n=6). Means with similar symbols do not differ significantly. Group I: Normal control group; Group II: Cyclophosphamide-induced control group; Group III: Alpha-lipoic acid-treated group; Group IV: Omega-3 fatty acids treated group; Group V: Alpha-lipoic acid+omega-3 fatty acids treated group.

Cyclophosphamide significantly (p<0.05) increased the average length of the estrous cycle (Figure-1) along with persistent diestrous (Figure-2). The estrous cycle in groups treated with alpha-LA, omega-3 fatty acids, and combination of alpha-LA+omega-3 fatty acids was significantly (p<0.05) regularized in terms of length of the entire cycle and duration of each phase (Figures-1 and 2). The group treated with combination therapy did not, however, demonstrate significant superiority when compared to monotherapies (Figures-1 and 2).

### Alpha-LA and omega-3 fatty acids normalized the hormonal imbalance in cyclophosphamide-induced rats

Cyclophosphamide demonstrated significant (p<0.05) increase and decrease in the levels of FSH and estradiol concentration, respectively. The groups treated with alpha-LA, omega-3 fatty acids, and the combination of alpha-LA+omega-3 fatty acids significantly (p<0.05) normalized the hormonal levels with no significant difference between the combination therapy and monotherapies (Table-1).

**Table-1:** Evaluation of clinical parameters in normal control and treated groups.

Groups (→)	Group I	Group II	Group III	Group IV	Group V

Parameters (↓)					
Follicle-stimulating hormone (mIU/ml)	2.88±0.23*	5.42±0.31^#^	2.18±0.28*	2.44±0.19*	2.96±0.14*
Estrogen (pg/ml)	37.4±2.32*	15.76±1.16^#^	39.13±2.24*	32.45±2.13*	35.12±2.21*
Reduced glutathione (nmol/mg protein)	9.45±0.54*	3.33±0.12^#^	8.54±0.59*	9.28±0.65*	10.06±0.87*
TNF-alpha (pg/ml)	27.89±2.21*	56.34±3.45^#^	24.33±1.87*	26.81±2.23*	28.23±2.05*

Values are expressed as mean±standard error of mean (n=6). Row means with different superscripts differ significantly (p<0.05). TNF-alpha=Tumor necrosis factor-alpha

### Alpha-LA and omega-3 fatty acid prevented oxidative stress and inflammation in the ovaries of the cyclophosphamide-induced rats

Cyclophosphamide significantly decreased (p<0.05) the activity of GSH and significantly (p<0.05) increased the concentration of TNF-alpha in the ovarian tissues. The groups treated with alpha-LA, omega-3 fatty acids, and the combination of both significantly (p<0.05) prevented oxidative stress and inflammation in the ovarian tissues by enhancing the activity of GSH and reducing the concentration of pro-inflammatory cytokine TNF-alpha. There was no significant difference between the treatment groups (Table-1).

### Alpha-LA and omega-3 fatty acid preserved the ovarian histological architecture in cyclophosphamide-induced rats

**Figure-3:**
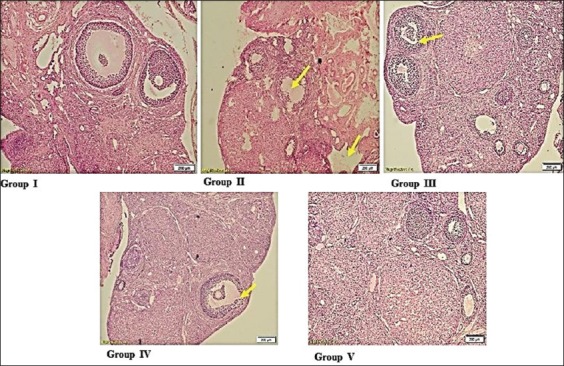
Group I (normal control group) showed normal architecture of ovaries; Group II (cyclophosphamide-induced control group) showed severe degeneration of granulosa cells and vacuolar degeneration of cytoplasm; Group III (alpha-lipoic acid-treated group) showed mild-to-moderate degenerative changes in ovaries; Group IV (omega-3 fatty acids treated group) showed mild pyknosis and vesicular degenerative in the ovaries; Group V (alpha-lipoic acid+omega-3 fatty acids treated group) showed normal architecture ovaries with mild pyknosis.

**Figure-4:**
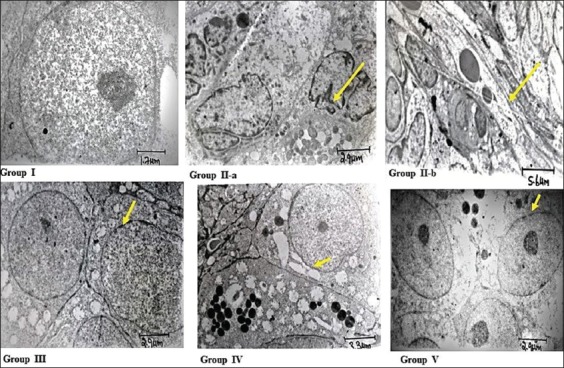
Group I (normal control group) showed normal architecture of ovaries follicular cytostructure (1000×); Group II-a (cyclophosphamide-induced control group) showed apoptotic bodies surrounded by fibrous (1500×); Group II-b (cyclophosphamide-treated group) shrunken cells, cortical fibrosis, and pyknotic nucleus (1500×); Group III (alpha-lipoic acid-treated group) intact nucleus, normal intercellular junction, mild cortical fibrosis, mild nuclear margination, and less amount of electron-dense material (1000×); Group IV (omega-3 fatty acids treated group) showed intact nucleus, normal intercellular junction, mild cortical fibrosis, mild nuclear margination, and less amount of electron-dense material (1000×); Group V (alpha-lipoic acid+omega-3 fatty acids treated group) showed normal architecture, intact nuclear membrane, and chromatin material with few electron-dense bodies (1000×).

Histopathological evaluation of the ovaries treated with cyclophosphamide alone revealed severe degeneration of granulosa cells with pyknotic nuclei, severe vacuolar degeneration of cytoplasm coupled with apoptotic bodies, and shedding of cells into the lumen. Histopathology of alpha-LA and omega-3 fatty acids as monotherapies and combination therapy revealed mild degenerative changes and otherwise normal architecture of the ovarian follicles comparable to the normal control (Figure-3). The transmission electron microscopy of the ovarian tissues treated with only cyclophosphamide revealed severe apoptosis surrounded by fibrous tissue, cortical fibrosis, and pyknotic nuclei. However, the group treated with alpha-LA showed uniform group of cells, distinct nuclear membrane with mild apoptosis, and few electron-dense structures, whereas the group treated with omega-3 fatty acids revealed intact nucleus, normal intercellular junctions with mild cortical fibrosis and nuclear margination, as well as less amount of electron-dense material. The group treated with the combination of alpha-LA and omega-3 fatty acids revealed normal architecture with intact nuclear membrane and chromatin material (Figure-4).

## Discussion

Cyclophosphamide-induced ovarian failure tremendously affects the quality of life in women undergoing chemotherapy. Cyclophosphamide is one of the most potent chemotherapeutic drugs that carry the highest risk for female infertility [54-56]. The toxic metabolites of cyclophosphamide cause oxidative stress and disruption of the intracellular antioxidant systems, leading to apoptosis of the ovarian follicles. Continuous exposure to cyclophosphamide causes degeneration of granulosa cells and rapid depletion of oocyte reserve subsequently, leading to ovarian atrophy [57]. The reactive metabolite of cyclophosphamide “phosphoramide mustard” binds to the DNA at the guanine position to form G-NOR, G-NOR-OH, and G-NOR-G adducts [58]. This DNA crosslinking leads to oxidative stress and disruption of antioxidant defense mechanisms, especially GSH concentration. The reactive free radicals generated as a result of oxidative stress destroy the rapidly dividing granulosa cells by triggering inflammation followed by apoptosis [58]. Thus, the gonadal steroid or the estradiol levels are reduced as a result of continuous damage to the granulosa cells of the ovarian follicle as the granulosa cells are virtually the only source of estradiol. This destruction of the granulosa cells and a decrease in gonadal steroid secretion, in turn, stimulates the hypothalamus to release GnRH through a negative feedback mechanism. The decrease in estrogen levels stimulates negative feedback to the hypothalamus, which, in turn, releases FSH to increase the recruitment of primordial follicles. The primordial follicles, on maturation, are again attacked by cyclophosphamide, leading to a vicious cycle of degeneration of granulosa cells and eventually apoptosis [59]. This pathology is clinically manifested by persistent elevation of FSH levels and reduced estrogen levels [60]. The ovarian follicular toxicity causes malfunctioning of the hypothalamic-pituitary-ovarian activities prolonging the estrous cyclicity with persistent diestrous [61-64].

Thus, from the above-mentioned mechanism, it is evident that cyclophosphamide exerts its toxicity through oxidative damage and impairing the antioxidant defense mechanism. This triggers the production of pro-inflammatory cytokines TNF-alpha that aid the process of apoptosis [11,65,66]. The findings of our study have corroborated with the previously reported toxicodynamics of cyclophosphamide, wherein animals treated with cyclophosphamide alone demonstrated a significant increase in oxidative stress and degenerative changes in the ovaries, irregular estrous cycles with persistent diestrous, and reduced GSH concentration coupled with increased levels of pro-inflammatory cytokine TNF-alpha.

From the parameters evaluated in the present study, we observed that alpha-LA and omega-3 fatty acids as monotherapies as well as in combination were effective in preventing cyclophosphamide-induced oxidative stress in the ovaries. However, an appreciable synergistic effect on administration of combination therapy was not observed. Further, in-depth evaluation to identify the synergistic effect of this combination was not performed and hence is not in the scope of the present study. However, we assume that specific *in vitro* studies pertaining to synergism and *in vivo* pharmacokinetics of the combination could help in elucidating the possibility of synergism.

The attenuation of the reproductive toxicity in the animals treated with alpha-LA and omega-3 fatty acids is attributed to the antioxidant properties of these compounds. Alpha-LA is converted to DHLA by lipoamide dehydrogenase. The LA/DHLA redox couple regenerates the endogenous antioxidants such as Vitamin C and Vitamin E and maintains the cellular GSH levels by transcriptional induction of genes responsible for GSH synthesis as well as neutralizes reactive oxygen and reactive nitrogen species, thus maintaining oxidant-antioxidant balance [17]. Regularization of estrous cycle by alpha-LA post-disruption by harmful chemicals such as cyclophosphamide has been previously reported [67]. On the other hand, omega-3 fatty acids are known to prevent the degradation of membrane phospholipids and subsequently cumulative oxidative stress. Thus, omega-3 fatty acids, being a precursor for important regulators of reproductive processes such as prostaglandins, thromboxanes, leukotrienes, and hydroxy fatty acids, are capable of regularizing estrous cycle and improve reproductive functioning [68].

## Conclusion

Fear of drug interactions and immunocompromised conditions limits the use of pharmacological interventions to prevent infertility associated with chemotherapy in cancer patients. In such a situation, nutritional intervention appears to be a safe bench side to bedside alternative to prevent infertility associated with chemotherapeutic agents. Based on the findings of the present investigation, dietary supplementation of alpha-LA and omega-3 fatty acids may help in ameliorating the severity of infertility associated with cyclophosphamide. However, further in-depth research to elucidate the pharmacodynamics of the antioxidants and substantiated clinical trials is essential to prove their effectiveness in cancer patients.

## Authors’ Contributions

DVN: The full experimental idea, planning, and design. Conduct of the experimental procedures, statistical analysis, and manuscript writing. MUR: Guidance in planning the experiment, interpretation of results, and manuscript revision. AGR: Guidance in planning the experiment and manuscript revision. BKK: Guidance in planning the experiment, interpretation of results, and manuscript revision. MAR: Assisted in conducting *in vivo* experimental procedures. ML: Provided guidance and expert advice on pathology aspects of the study. UR: Provided guidance and expert advice for the analysis of molecular parameters in the study. All authors read and approved the final manuscript.
